# A role for qualitative methods in researching Twitter data on a popular science article's communication

**DOI:** 10.3389/frma.2024.1431298

**Published:** 2025-01-07

**Authors:** Travis Noakes, Corrie Susanna Uys, Patricia Ann Harpur, Izak van Zyl

**Affiliations:** ^1^Faculty of Health and Wellness Sciences, Cape Peninsula University of Technology, Cape Town, South Africa; ^2^Applied Microbial and Health Biotechnology Institute, Cape Peninsula University of Technology, Cape Town, South Africa; ^3^Department of Information Technology, Faculty of Informatics and Design, Cape Peninsula University of Technology, Cape Town, South Africa; ^4^Centre for Postgraduate Studies, Cape Peninsula University of Technology, Cape Town, South Africa

**Keywords:** content analysis, debate about health science, microblogging data, multimodal content analysis, research method, semantic network analysis, science communication, small data analysis

## Abstract

Big Data communication researchers have highlighted the need for qualitative analysis of online science conversations to better understand their meaning. However, a scholarly gap exists in exploring how qualitative methods can be applied to small data regarding micro-bloggers' communications about science articles. While social media attention assists with article dissemination, qualitative research into the associated microblogging practices remains limited. To address these gaps, this study explores how qualitative analysis can enhance science communication studies on microblogging articles. Calls for such qualitative approaches are supported by a practical example: an interdisciplinary team applied mixed methods to better understand the promotion of an unorthodox but popular science article on Twitter over a 2-year period. While Big Data studies typically identify patterns in microbloggers' activities from large data sets, this study demonstrates the value of integrating qualitative analysis to deepen understanding of these interactions. In this study, a small data set was analyzed using NVivo™ by a pragmatist and MAXQDA™ by a statistician. The pragmatist's multimodal content analysis found that health professionals shared links to the article, with its popularity tied to its role as a communication event within a longstanding debate in the health sciences. Dissident professionals used this article to support an emergent paradigm. The analysis also uncovered practices, such as language localization, where a title was translated from English to Spanish to reach broader audiences. A semantic network analysis confirmed that terms used by the article's tweeters strongly aligned with its content, and the discussion was notably pro-social. Meta-inferences were then drawn by integrating the findings from the two methods. These flagged the significance of contextualizing the sharing of a health science article in relation to tweeters' professional identities and their stances on health-related issues. In addition, meta-critiques highlighted challenges in preparing accurate tweet data and analyzing them using qualitative data analysis software. These findings highlight the valuable contributions that qualitative research can make to research involving microblogging data in science communication. Future research could critique this approach or further explore the microblogging of key articles within important scientific debates.

## Introduction

Since 1997, social media platforms have presented new opportunities for academic experts to engage in two-way communication with peers and other networked publics. The networked public sphere (Benkler, 2006) encompasses all spaces that enable the formation of online public discourse. These may be connected through users (in both content production and consumption), signals such as hyperlinks, or shared content. Scholars increasingly use popular digital platforms such as LinkedIn and Twitter (now called X)^1^ to promote their latest publications and build their reputations. This “pushed” dissemination of scholarship to the networked public differs from traditional academic dissemination. Conventional journal articles and conferences rely on readers pulling their information from a knowledge base that is typically restricted to users from subscribing to academic institutions.

This study examines the dynamics of sharing scientific articles on Twitter, highlighting its role as a platform for science communication and public engagement. This popular microblogging platform has between 550 and 360 million monthly active users worldwide. Micro-bloggers create short messages for real-time communication. This genre's immediate nature supports its users with staying up-to-date on current events. Scholars post on microblogs to communicate online events linking to their publications, encouraging networked discussions on state-of-the-art research.

The importance of such public engagement is recognized through the emergence of *altmetrics*. Its “alternative bibliometrics” complements traditional citation-based metrics by adding social media outreach to the quantitative analysis of scholarly output and publication (Priem et al., 2012). Altmetrics monitors the sharing of research publications on social media, reference managers, scholarly blogs, and mass media coverage (Moed, 2016). Within popular social network sites, traces of users' deliberations, conversations, and amplification of research articles are tracked. Altmetrics mirrors how network users' actions become a *Big Data* by-product. This involves the algorithmic processing of extensive digital footprints, or “data traces,” representing user activities on various platforms (Latzko-Toth et al., 2017). *Altmetric* reports provide insights into the online attention that scholarly documents receive (Fraumann, 2017) and the impact of policy research (Fang, 2021). Twitter metrics for research publication amplification are the most popular data sources that provide the basis for altmetrics (Haustein, 2019).

In science communication (SciComm), quantitative researchers have employed Big Data to investigate the sharing and amplification of science articles on microblogging networks. Related topics that such scholarship explores have included how Twitter users' activity around research publications can be characterized (Díaz-Faes et al., 2019), scientists' sharing of article links (Maleki, 2014), the relationship between Twitter altmetric results and citation results (Costas et al., 2015), the increasing value that academic journals see in promoting their articles on Twitter (Erskine and Hendricks, 2021), the quality of engagement from Twitter audiences with scientific studies (Didegah et al., 2018), and how articles are referenced in Twitter conversations, as part of broader argumentative patterns (Foderaro and Lorentzen, 2023).

SciComm researchers exploring microblogging conversations have called for the use of qualitative approaches that might add new insights regarding digital discourse related to science article shares (Foderaro and Lorentzen, 2023; Lorentzen et al., 2019; Nelhans and Lorentzen, 2016). Including qualitative research approaches inevitably entails a shift to *small data projects* since these do not use the proliferation of digital traces that Big Data ones do. This approach allows for detailed analysis using manual methods. Small data projects focus on data provided in tightly controlled ways using sampling techniques that limit their scope, temporality, and size (Kitchin and McArdle, 2016). An emergent approach, “small data,” combines basic quantitative metrics with a close reading of the selected microblogging data (Stephansen and Couldry, 2014).

Researchers have described how small data approaches can complement other qualitative methods, such as interviews (Latzko-Toth et al., 2017). However, for researchers focused solely on using small data to study microblogging, no specific rationale is available for qualitative research's potential contribution. This gap starkly contrasts with how qualitative methods for studying social media communication are well-established in their support for a fuller understanding of the context of media practices (Boyd and Crawford, 2012; Quan-Haase et al., 2015). Such methods support a fuller description of the social context of scientific research (Allen and Howell, 2020), plus how practices for a scientific article's sharing may be linked to agreement or dissent in broader science controversies (Venturini and Munk, 2021). The sharing of articles depends on the individual, making it worthwhile for researchers to situate how such practices relate to individuals' identity work. Health practitioners present themselves online by following the strategies of experts. These range from describing their credentials to active listening and making referrals (Rudolf von Rohr et al., 2019).

To address the missing rationale, this study sought to answer the question:

*RQ. What role do qualitative methods play in researching Twitter data for a popular science article's sharing?*

This question addresses a gap in the literature. Communication research into scientific Twitter often employs Big Data approaches, characterized by large sample sizes. Without a clear rationale for the value of small data approaches, the potential contribution of qualitative research to understanding microblogging communications risks being overlooked. As Borgman (2015) suggests, data can be understood as “big” or “little,” depending on how they are analyzed and utilized. Small approaches, when appropriately scaled to the phenomenon of interest, can still yield significant insights. This article provides a practical example, demonstrating distinctive insights derived from two qualitative lenses applied to the sharing of a science article on Twitter.

The Introduction grounds the salience of this study's research question by first tackling the emergence of new digital genres for science dissemination. Subsequently, the study addresses the importance and use of the “Scientific Twitter” genre. Finally, the introduction calls for qualitative analysis of microblogging data related to scientific conversations.

### Emergence of new genres for science communication

The emergence of Web 2.0 technologies enabled the networked public to exercise public voice, such as students who become online content creators (Brown et al., 2016). New communication formats (“TikToks,” retweets) serve a wide range of communicational purposes, from entertainment to deliberation on scholarship. Simultaneously, the emergence of new content genres promotes online communication, e.g., Reddit science discussion boards (Pflugfelder and Mahmou-Werndli, 2021). Science communication has witnessed the evolution of ScienceTok, post-peer-review, and *Scientific Twitter*—the focus of this study. Scientific tweeting is a new science communication genre (Weller et al., 2011), in which scientists microblog about the successes and failures in their fieldwork and ask for advice (Bonetta, 2009). Scholars microblog to share their findings, deliberate on science topics, and stay abreast of the literature. Researchers may microblog about careers, grants, science policy, and other issues. As such, this genre of science popularization is recognized as a new form of scientific output that shares characteristics with other forms of scientific discourse (Costas et al., 2015).

### Leveraging of Twitter as a communication platform of choice

The scientific Twitter genre is non-trivial due to its popularity with researchers: Twitter has been one of the most popular digital platforms among researchers because it supports open engagement with science (Cormier and Cushman, 2021). Over 290,000 scholars on World of Science and Altmetric.com have Twitter accounts (Costas et al., 2020). Scientists who used it potentially comprised 1–5% of its 187 million user base in 2017 (Costas et al., 2017). Between August 2011 and October 2017, over 3.5 million unique Twitter profiles shared tweets that included scholarly output (Díaz-Faes et al., 2019).

Twitter's qualities as a digital platform that is open by default, cheap to access, quick to learn via “texting,” and easy to use contributed to it becoming ubiquitous (Murthy, 2018). Twitter has emerged as the microblog of choice for scientists to communicate with like-minded peers, members of the public, organizations, and the media (Collins et al., 2016). Scholars in academic institutions use Twitter to showcase their professional expertise (Vainio and Holmberg, 2017), connect with colleagues, and share peer-reviewed literature (Priem and Costello, 2010) and self-authored content. They tweet to educate others (Noakes, 2021), follow article discussions, add commentary (Van Noorden, 2014), participate in asynchronous journal clubs (Chary and Chai, 2020), and critique articles almost immediately after publication (Mandavilli, 2011; Yeo et al., 2017). Such communal feedback can complement traditional peer review methods, providing an additional layer of engagement (Sarkar et al., 2022). Scientists use Twitter to engage in discussions with science policymakers (Kapp et al., 2015) and to live-tweet during conferences (Kapp et al., 2015; Collins et al., 2016).

Scientific institutions use Twitter to promote events such as festivals (Su et al., 2017), while its features also facilitate multilingual and EFL (English as a Foreign Language) communication in virtual academic conferences (Márquez and Porras, 2020). Science organizations use hashtags and other affordances for their community-building practices (Su et al., 2017).

Twitter is a popular data source for health-related research, ranging from professional education in healthcare to big data and sentiment analysis (Yeung et al., 2021). As a communication tool, Twitter supports the exploration of the outcomes of divergent styles of science communication, such as the efficacy of humor in tweets (Yeo et al., 2020) and how types of humor are associated with retweets, likes, and comments (Su et al., 2022). Scientists joke about their work using the satirical hashtag #overlyhonestmethods (Simis-Wilkinson et al., 2018).

Limited research has focused exclusively on microblogging conversations in the sharing of science articles (Foderaro and Lorentzen, 2023). Few quantitative studies have addressed this (Nelhans and Lorentzen, 2016). Researchers have explored users whose science tweets link to articles (Maleki, 2014); patterns in Twitter conversations on health (Ola and Sedig, 2020); demonstrating a method for studying the use of scientific sources on Twitter (Foderaro and Lorentzen, 2022); and the argument stages in climate change threads (Foderaro and Lorentzen, 2023).

### Exploring online science conversations with qualitative methods

This study responds to calls for qualitative analysis of Twitter data in science-related conversations and arguments (Lorentzen et al., 2019; Nelhans and Lorentzen, 2016; Pearce et al., 2019). Quantitative researchers have shown that extensive conversations about research can be collected from Twitter. This scientific dialogue is suitable for various automated analysis methods. Since such analysis cannot say much about how Twitter users interact, a need to contextualize conversations via qualitative methods was identified:

Pearce et al. (2019) conducted a systematic and critical literature review focusing on discussions regarding climate change via social media. The review found a substantial bias toward Twitter studies. Gaps were identified regarding qualitative analyses, studies of visual communication, and alternative digital platforms to Twitter. In response to this call, Foderaro and Lorentzen (2023) investigated the practices of argumentation on Twitter by collecting conversational threads focused on climate change. They highlighted that little scholarly attention has been given to interactions in a conversational context. Their content analysis applied coding to tweets at different stages of argument and how linked and embedded sources were used.

Additionally, the plausibility and soundness of a message were coded, alongside the consistency and trustworthiness of linked sources, plus adequacy for the target audience. Foderaro and Lorentzen found that arguing parties were unable to convince each other even with reasonable arguments. This outcome was attributed to the absence of shared values or common premises among participants (p. 145). Consequently, it was more important for Twitter conversationalists to convince their audiences rather than change their opponents' minds.

Another example of research into Twitter conversation undertaken by Lorentzen et al. (2019) analyzed scientific study links. Information retrieval techniques compared segments of these conversations to locate differences and similarities between and within discussions. A qualitative analysis of tweet practices explored the use of unusual terms and categorized distinct conversational properties and academic-linking styles. The authors identified a need to identify controversial issues discussed on Twitter with more sophisticated machine-learning approaches or via qualitative methods. Lorentzen et al. recommended studying complete conversations to better explain how people interact on Twitter.

Qualitative research can address such communications' meanings and their relationship to micro-bloggers' identity work in presenting themselves in the way they do. The calls from SciComm scholars resonate with those from the field of digital discourse analysis. Its researchers are urged to move their field forward by studying the relational practices in social media, users' identity work, and sociability (Garcés-Conejos Blitvich and Bou-Franch, 2019).

## Background literature

The authors' literature review process focused on the role that qualitative methods might play in understanding microblogging practices, with the article being shared in the health sciences field.

### Research into health science content promotion on Twitter (or X)

Several studies address article sharing and amplification via Twitter. These drew on quantitative or mixed-methods methods to research how epidemiologists exploit the emerging genres of Twitter for public engagement (Tardy, 2023); Twitter (and Facebook and Instagram)'s use by eye-specialist journals, professional societies, and organizations (Cohen and Pershing, 2022); how surgeons use social media platforms (including Twitter) for research communication and impact (Grossman et al., 2021), likewise for biomedical scientists, and how their microblogging choices influence their followings (Sarkar et al., 2022).

Scholars have characterized the landscape of precision nutrition content on Twitter, with a specific focus on nutrigenetics and nutrigenomics (Batheja et al., 2023), and explored how sharing visual abstracts promotes wider suicide prevention research dissemination and altmetrics engagement (Hoffberg et al., 2020). Researchers have explored the anti-vaccination movement's referencing of vaccine-related research articles (Van Schalkwyk et al., 2020), plus changes in eight COVID-19 conspiracy theory discussions over time (Erokhin et al., 2022).

Regarding scholarship on the promotion of health science articles, scholars have described a year-long scientific Twitter campaign, #365Papers, that tweeted one peer-reviewed publication related to cancer and exercise/physical activity per day (Zadravec et al., 2021). Findings suggested this daily campaign stimulated peer and public engagement and dialogue around new scientific publications. Incorporating prominent research field figures into the campaign process assisted strongly with its outreach. A study into the short-term impact of a #TweetTheJournal social media promotion for select open-access psychological journals' articles found that the campaign resulted in a statistically significant, higher Altmetric attention score (Ye and Na, 2018). Researchers have compared the user engagement performance of articles that the Cell journal posted on Twitter and Facebook. An examination of 324 posts suggested that user engagement positively impacted article visits (Cui et al., 2023). Research into stroke-related journals' Twitter usage found that their more frequently tweeted articles tended to have higher citation rates (Sousa et al., 2022). An analysis of 110 articles from PeerJ found that while social media attention follows publication, it does not last long (Cui et al., 2023). This finding resonated with Zhang et al. (2023)'s articles representing recent scientific achievements, which may be tweeted for longer after catching society's attention.

While the social media sharing of health science articles can have a positive influence on their dissemination and citation rates, no research examples could be found that solely focused on specific articles' Twitter communication or proposed a rationale for the role of qualitative research with microblogging data.

## Materials and methods

Lacking an exemplar to follow, this study developed a qualitative-led approach to grow an understanding of Twitter communications around a scientific article. The six-phased research process comprised: (i) selecting an article to focus on from a long-running scientific debate; (ii) identifying what hyperlinks related to the article could be shared on Twitter; (iii) importing tweet shares into NVivo™ and benchmarking them vs. altmetrics, (iv) preparing a codebook and coding the data in NVivo™; (v) importing the conversations into MAXQDA™ for coding; and (vi) comparing a multimodal content- and semantic network analysis to discuss meta-inferences.

### Selecting a popular article suitable for qualitative analysis

A long-standing academic debate in the health sciences contrasts proponents of the 'cholesterol' model (CM) with those advocating an alternative insulin resistance (IR) paradigm. They argue for an IR paradigm of chronic ill health and low-carbohydrate, healthy fat (LCHF) lifestyles (Noakes et al., 2023b). South Africa has seen many communication episodes involving leading scholars in this debate (http://bit.ly/2OSMfUx). In November 2019, the researchers selected a popular article from among these episodes. Its small dataset of users and tweets seemed feasible for manual analysis via qualitative data analysis software (QDAS). This article (Webster et al., 2019) illuminated how 24 South Africans put their diabetes in remission by following an LCHF diet sustainably with minimal support from health professionals. In February 2024, Digital Science's Altmetric platform showed that this study had achieved an *attention score* of 93. This placed it among the top 5% of all research outputs Altmetric calculates (Altmetric, 2021). On X, the article had been featured in 158 posts from 137 users, with an upper bound of 632,585 followers.

### Identifying which article hyperlinks could be shared on Twitter

**Figure 1:**
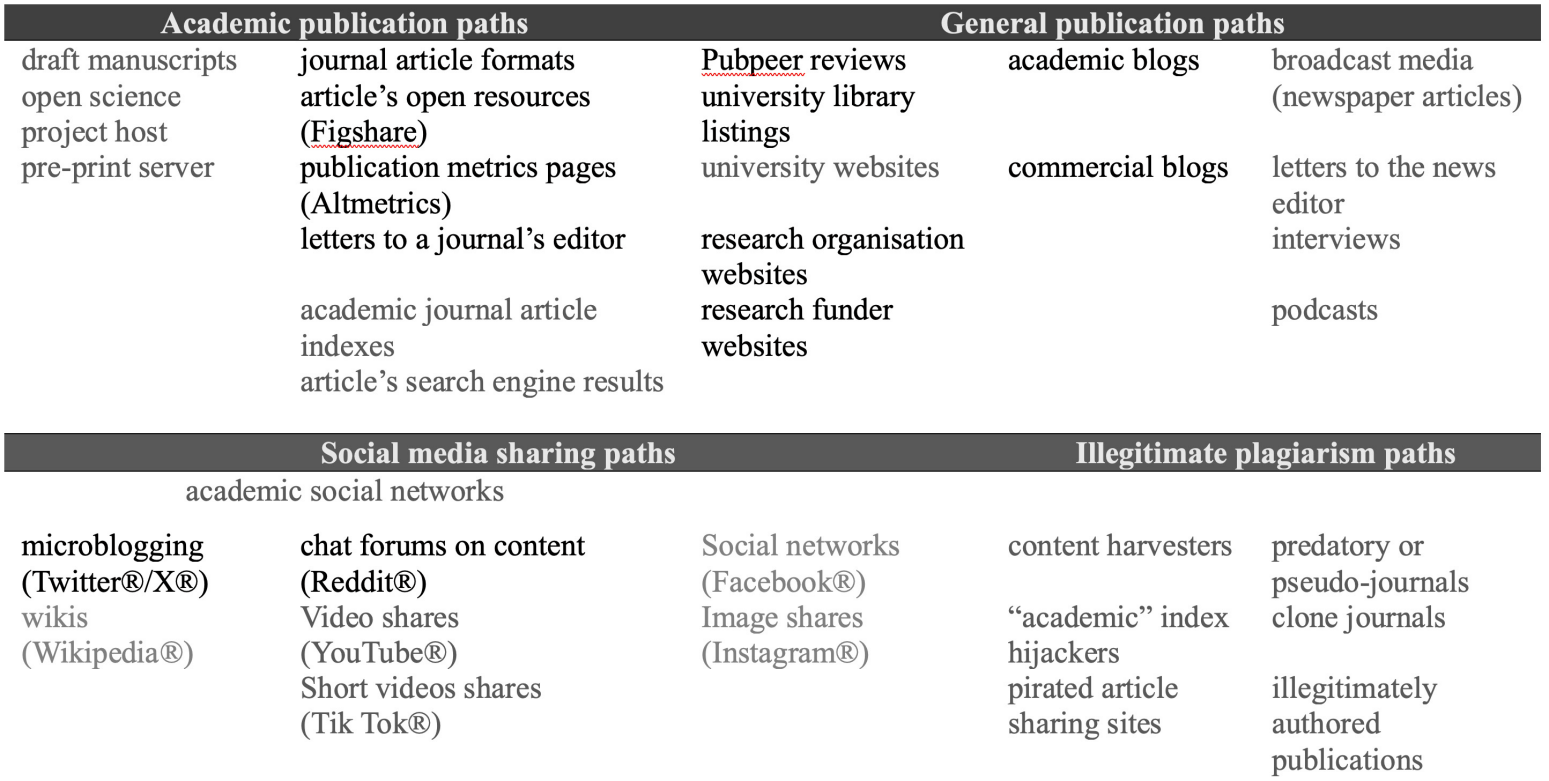
Diverse academic publications that might feature in a journal article science communication event.

An iterative process was followed to identify the publication links that might be shared (see Figure 1) via Twitter in relation to Webster et al. (2019). These were divided into shares from (i) academic publishers and (ii) other sources.

Under (i), micro-bloggers shared a DOI link, three Dovepress URLs, and two PubMed URLs. Six URLs under (ii) included a critical letter, an author's reply, two blog posts, and Reddit forum discussions. Data extracts were run by Younglings Africa in April 2022 for (i) and (ii), which produced a spreadsheet for each hyperlink's shares. These started from the article's digital publication (5th of December 2019) up to March 2021. This data was extracted the following month.

Quantitative researchers have flagged pertinent concerns regarding flawed science communication on Twitter, plus related limitations in assuming that tweet counts used in Altmetrics denote valuable public outreach (Robinson-Garcia et al., 2017). This study followed their recommendations for removing mechanical (re)tweets while focusing on original tweets linked to science article shares. The analysts checked Twitter users' account activities, plus linked profiles, to ensure they were genuine, not Twitter bots. Automated “contributors” to Scientific Twitter are a real threat in skewing Altmetrics results (Arroyo-Machado et al., 2023).

### Importing X data into NVivo™ and benchmarking formal shares

For accurate and comprehensive tweet data, the research team relied on the extraction code that queried Twitter's API. It also provides data provision services (Bruns and Burgess, 2016). To double-check the accuracy of Webster et al.'s (2019) sharing, (i) extractions were benchmarked against the Altmetric results. This revealed that a few tweets had not been extracted. Webster et al. (2019)'s lead author had deactivated his Twitter account. Deletion of accounts and their tweets is the main reason for scientific publication mentions becoming unavailable, followed by the suspension or protection of tweeters' accounts (Fang et al., 2020). Another issue was that data extractions did not mention any URL in replies to retweets or longer versions of a URL being used. These “missed tweets” were manually captured in a dedicated spreadsheet. Following its import into NVivo™ for Mac, the file's tweets and user account information matched the early March Altmetrics report for Twitter shares (Digital Science, 2022).

### Refining the multimodal content analysis' codebook

Social semiotics is an approach to communication that strives to understand how people communicate by various means in specific social settings (Hodge and Kress, 1988). A social semiotic multimodal content analysis focuses on how people make signs in the context of interpersonal and institutional power relations to achieve specific aims. Modes of communication offer historically specific and socially and culturally shared options for communicating or *semiotic resources*. Researchers have analyzed how such resources are used in microblogging. Michele Zappavigna analyzed Twitter posts to explore ambient affiliation in expressions of self-deprecation, addiction, and frazzled parenting (Zappavigna, 2014). Her research demonstrated that identities can be thought of as bonds when approached in terms of the social relations they enact. Identities might be regarded as patterns of values when considered in terms of the meanings that they negotiate in discourse.

The semiotic resources that tweeters of Webster et al. (2019) used (such as @mentions, # hashtags, types of URL, and article screengrabs) were defined in the pragmatist's codebook. It was iteratively refined in response to interpretation needs and new literature. Coding the data revealed surprising interpretation challenges. The concept of “level of tweet reply” was added to easily locate a tweet's order in a thread. This ranged from first, as a “*deliberation*,” to being the “*15th reply to a 14th tweet*,” An example of the ongoing literature review informing needed coding changes concerned the schema from Nelhans' *Twitter conversation patterns related to studies* (Nelhans and Lorentzen, 2016, p. 28–9). Its outline was adopted to remedy missed codes and for labeling refinement.

### Semantic network analysis of Twitter

Scholars should integrate different analytical methods for a more comprehensive understanding of Twitter discussions (Dai and Higgs, 2023). A statistician completed a semantic network analysis (SNA) to add to understandings derived from the pragmatists' work. This approach has been well-used for studying the discourse surrounding social movements and public health crises on microblogging platforms. SNA can offer novel perspectives regarding the structural relationships and meanings embedded within tweet content. SNA is a word analysis that explores the proximity of words in a text, whether in pairs or groups. In relation to Webster et al.'s (2019) shares, the SNA spotlighted the semantic connections between words, topics, and users in this data. This was intended to support insights into how the digital public disseminated, discussed, and perceived the article. Understanding the influential actors within this network and how they framed their messaging and interacted with others in discussing the prevalent themes.

There is a wide variety of SNA approaches, but for this study, the statistician's approach was process-driven in working with static Twitter data in MAXQDA™ for the first time. This involved becoming familiar with the many microblogging data fields in QDAS and how codes for content and user information could overlap. The pragmatist shared his codebook and spreadsheet extracts, which the statistician imported into both MAXQDA™ and ATLAS.ti™. For the first SNA step (S1), she created a visual word cloud view of the quantitative data. The next step (S2) explored the proximity of all codes, which proved sufficient for the analysis.

To prepare (S1) word cloud visualizations, the statistician initially searched for words to auto-code by doing a word frequency map in MAXQDA™. These results were checked against ATLAS.ti's, and they matched. All words that featured, whether from profiles or tweet content, were then sampled to produce a word cloud of the most popular terms. This defined prevalent topics within that article's sharing network. Subsequently, the statistician developed a (S2) proximity of codes map. She explored how closely the codes occurred to each other by coding co-occurrences and exploring their proximity visually through network maps. MAXQDA™ displayed the codes according to their “close instance,” how closely they feature together. Each SNA step involved an interplay between quantitative and qualitative approaches. While the steps primarily relied on quantitative analysis, interpreting the visual maps required qualitative insights. Overall, the process was qualitatively driven, with the analyst acting as a “research instrument” to identify the most salient codes.

### Generating meta inferences

The final phase involved a comparison of the multimodal content and SNA's results in a mixed-methods approach (Venkatesh et al., 2023). MAXQDA™ is a QDAS tool that supports a statistician's mixed methods with (live) Twitter data (Noakes et al., 2023a), while NVivo™ supports multimodal tweet analysis best. Both QDAS offer quantitative reporting options for either style of analysis. Though it focuses on the contributions of qualitative research, this study foregrounds its aspects from both lenses. This follows the recommendation of Schoonenboom and Johnson (2017) for a mixed-methods research approach to be qualitative dominant or qualitatively driven.

Genuine mixed-method projects conclude with a meta-inference that connects or integrates various claims (Schoonenboom, 2022). A meta-inference is an “overall conclusion, explanation, or understanding developed through an integration of the inferences obtained from the qualitative and quantitative strands of a mixed methods study” (Tashakkori and Teddlie, 2008, p. 102). A meta-inference and its internal structure are developed in successive steps of claim integration, whereby two or more simpler claims are integrated into one more complex claim (Schoonenboom, 2022).

In phase vi, the researchers sought to establish *meta-inferences* across the presented findings by following the ideal seven-step process. The first three involve defining separate inferences: (i) knowledge-based, (ii) experience-based, and (iii) data-driven, qualitative, and quantitative ones. The investigators then design (iv) inference association maps before (v) eliminating speculative inferences. (vi) Meta inferences are then generated and finalized, as the authors contrast claims to explore which were confirmatory, explanatory, or seemed to feature juxtapositions and contradictions. Step (vii) uses “working backward heuristics” as a meta-inference validation tool. Following the overall comprehensive process enables researchers to “*apply analytical, reflexive, and visual tools concurrently to make explicit how meta inferences are linked to the knowledge base, the researchers*” *experiences, and the actual qualitative and quantitative data from the participants* (Younas et al., 2023, p. 289).

## Evidence

In response to this study's research question, this section presents key findings from the pragmatist's and statistician's analyses:

### Key findings from a pragmatist's multimodal analysis

The study incorporated a social semiotic multimodal content analysis. The analysis produced four claims for the article's sharing. It was (M1) predominately shared by IR/LCHF proponents. In contrast, (M2) critics replied to their tweets. The article's sharers followed a (M3) myriad of practices in sharing links, and their conversational threads were (M4) pro-social.

### M1 is often shared by IR and LCHF health professionals

Most of the participants who shared and discussed the science article were health professionals (see Table 1). They typically present their occupations and related expertise online to establish credibility and trust (Sillence, 2010). The high number of profiles mentioning their “occupation” and “expertise” agreed with Vainio and Holmberg's (2017) finding that tweeters who share popularly-tweeted scientific articles tend to describe themselves with these two categories.

**Table 1:** Frequency of roles described in profiles, plus examples.

**Code**	**Role description**	**Example**	**References**
C1	Occupational roles		29
C1.1	Diet follower or nutritional advice	Health Coach #fasting #lowcarb	24
C1.2	Medical	MD (Occupational Medicine) | Health Professions Educationist	18
C1.3	Academic	Associate Professor of Exercise Science, Researcher	13
C1.4	Science-related	Love the #science behind life and #health	7
C1.5	Sports-related	Love anything related to running, nutrition, and exercise science	11
C1.6	Author	a talented writer is the author of #Keto #Recipes	6
C1.7	Business and PBO organizations	Our goal is to support the dietary revolution that will reverse the global epidemics of obesity and type 2 diabetes mellitus.	7
C2.1	Nationality	Just a regular Scot based in Oz.	2
C2.2	Politically related	Constitution following American citizen who refuses the narrative.....	3
C2.3	Online behaviors	I don't reply to bots + pseudonyms	7
C.2.3.1	Other digital accounts	Follow me on Mastodon (URL) or Telegram (URL)	4
C2.3.2	Personal views	… sometimes private thoughts.	3
C2.4	Other interests		10
C2.4.1	Family	proud mom	2
C2.4.2	Green/Environmental	Committed to Earth, decency, ecosystems, and sustainability.	4
C2.4.3	Personal style	Intellectually stimulating, acerbically witty, and socially confrontational. I will eventually offend you in some way.	1
C2.4.4	Pets	Love running, dogs, and camping.	1
C2.4.5	Technology	Gadget fanatic and proponent of free and open-source software.	1
C2.4.6	Vintage clothing	Vintage clothing is a passion!	1
C2.5	Self-parody	Venus flytrap glorious own goal	3
C3	Empty profile		2

Online commentators' profiles reflect the effect (Barnes, 2018), and many of the article's sharers' profiles took a stance that endorsed low-carbohydrate diets. Specific expressions for LCHF support ranged from lengthy self-descriptions (e.g., “40 years old but fitter and stronger than when I was 30 thanks to Keto, then carnivore for the last 3 years”) to the simple (“Unashamedly carnivore”) or via hashtags that ranged from the broad #LCHF movement to the specific #ketones. Four authors indicated that they had written pro-IR publications (ranging from capsules on various health topics in French at Dogmez-vous to blogposts for DietDoctor.com and two books—“Quick Keto” and “The Banting 7-Day Meal Plans”). Promotion of the LCHF lifestyle (or “diet”) denoted support for the IR paradigm, as its proponents believe that “(LCHF) food is medicine.” Just as “Medical” and “Diet follower or nutritional advice” role descriptions could denote IR support, so did “sports-related” ones. Mentioning belonging to a fitness chain for extreme conditioning program training could denote support for LCHF if that chain endorses LCHF as foundational to wellbeing.

It was unsurprising that publications for the IR paradigm were almost exclusively shared by users expressing support for the LCHF lifestyle (or “diet”). This matches Vainio and Holmbergs' finding that scientific articles are tweeted to promote ideological views, especially when an article represents a topic that divides general opinion (Vainio and Holmberg, 2017). As cultural sociology explains, when individuals classify objects, they simultaneously classify themselves (Bourdieu, 1986). LCHF proponents who shared the Webster et al. (2019) article earlier connoted being up-to-date with current IR research developments. Micro-bloggers' academic and scientific role mentions did not address the IR, or cholesterol, paradigm. Rather, “scholarly roles” focused on degrees and work achievements, while “science roles” foregrounded scientific goals, philosophies, and caveats on the Twitter content (“*Spreading scientific information, not medical advice*”).

### M2 critics replied to tweets but did not share the “controversial” article

The authors and the funding organization behind their manuscript first shared links to Webster et al. (2019). Their deliberations soon attracted like-minded health professionals who supported IR interventions. In contrast, no deliberations from critics were found in the original article shares. The choice to tweet a science article's link is tied to online identity work since micro-bloggers follow narrativization processes in making aspects of themselves and their interests visible (Dayter, 2016; Sadler, 2021). This occurs in Twitter profiles, original posts, and in what tweeters reshare and like. Such construction of digital identity is not a static process but an ongoing and reflexive construction of selfhood (Cover, 2014). Promoting pro-IR articles via posts is congruent with LCHF proponents' ongoing digital identity work. By contrast, sharing evidence for an unconventional LCHF approach would question an orthodox health scientist's identity.

### M3 a myriad of link-sharing practices

Twitter users drew on a surprisingly wide variety of communication practices while sharing the research article's link. Table 2 shows their codes: Examples of academic shares included one as part of the reading list for a lecture and being linked from a conference presentation. Two participants in the article's study mentioned contributing their data. A few doctors shared the article for affirming their treatment protocols, as did nutritionists and dietitians. The article was linked to #WORLDdiabetes Day as part of an LCHF lifestyle promotion. The article's key point(s) were translated by Spanish and Portuguese health micro-influencers for their Twitter followers.

**Table 2:** Practices in communicating a research article's link.

**Index value in NVivo™ codebook**	**Deliberation's content**	**Mention in files**	**Total code references**
D1	Promoting a science article	4	15
D1.1	Affirmed for medical treatment	4	9
D1.2	Affirming low-carb lifestyle dietary choices	3	6
D1.3	Translation of the article's title or content	2	4
D1.4	Linking to events for healthy lifestyle promotion	1	1
D1.5	Invite other users to a (potential) discussion	2	9
D1.5.1	@-mention use	2	14
D1.6	Linking to # hashtag communities	2	6
D1.6.1	# hashtag use	2	6
D1.7	Promoting a post on the science article	2	2
D2	Use in scholarly contexts	4	8
D2.1	As part of a lecture–reading list	1	1
D2.2	Linked to a conference presentation	1	2
D2.3	Reply to an expert	1	1
D2.4	Participant in fieldwork	1	2
D2.5	Responding to a journal letter	1	2
D3	Promotion of related online content	1	1
D3.1	LinkTree	1	1
D3.2	Reddit	1	1
D4	Further than the research study's content	2	7

### M4 pro-social communication threads

A microblogging conversation begins with an original post and may branch into various threads through replies. These replies can lead to consensual, discursive, or confrontational exchanges (Barnes, 2018). Although the majority of Twitter discussions around Webster et al. (2019) did not generate replies, those that did were constructive and focused on the article's content. Conversations involved knowledgeable contributors, whether IR/LCHF proponents or their critics. Contributing to their genuine identities, such experts carried the formal norms for civil communication into the scientific Twitter genre. The consensual threads included a request for further information, an agreement marking a thread's conclusion, and praise for the article. The discursive threads featured a query on why Professor Noakes's response letter did not include references and when a rebuttal to his response might be expected from the original letter's writer. While there were relatively few confrontational threads, critics did emphasize the article's methodological limitations. A tweet claim that diabetes might be “reversed” was corrected with the observation that it is rather put in “remission.”

### Key findings from a statistician's semantic network analysis

The statistician developed six main claims from her SNA. The first (S1) confirmed that Twitter posts were strongly tied to Webster et al.'s (2019) focus on diet and diabetes. The second (S2) revealed academic links were shared repeatedly by prominent IR advocates. Finally, the (S3) most repeated shares came from England and South Africa. The fourth (S4) collaborated on the findings of the multimodal content analysis regarding who was involved and the content they shared. The fifth claim (S5) stemmed from a critique of the automated quantitative methods in QDAS inaccurately reporting results for “sentiment.” In response, qualitative-led studies could help accurately identify sentiment and users' stances. Since the researchers' close views of “clean” data flagged a few other concerns, finding (S6) concerned how qualitative approaches could add valuable meta-criticisms of Twitter data.

### S1 Twitter communications closely link to the article's content

**Figure 2:**
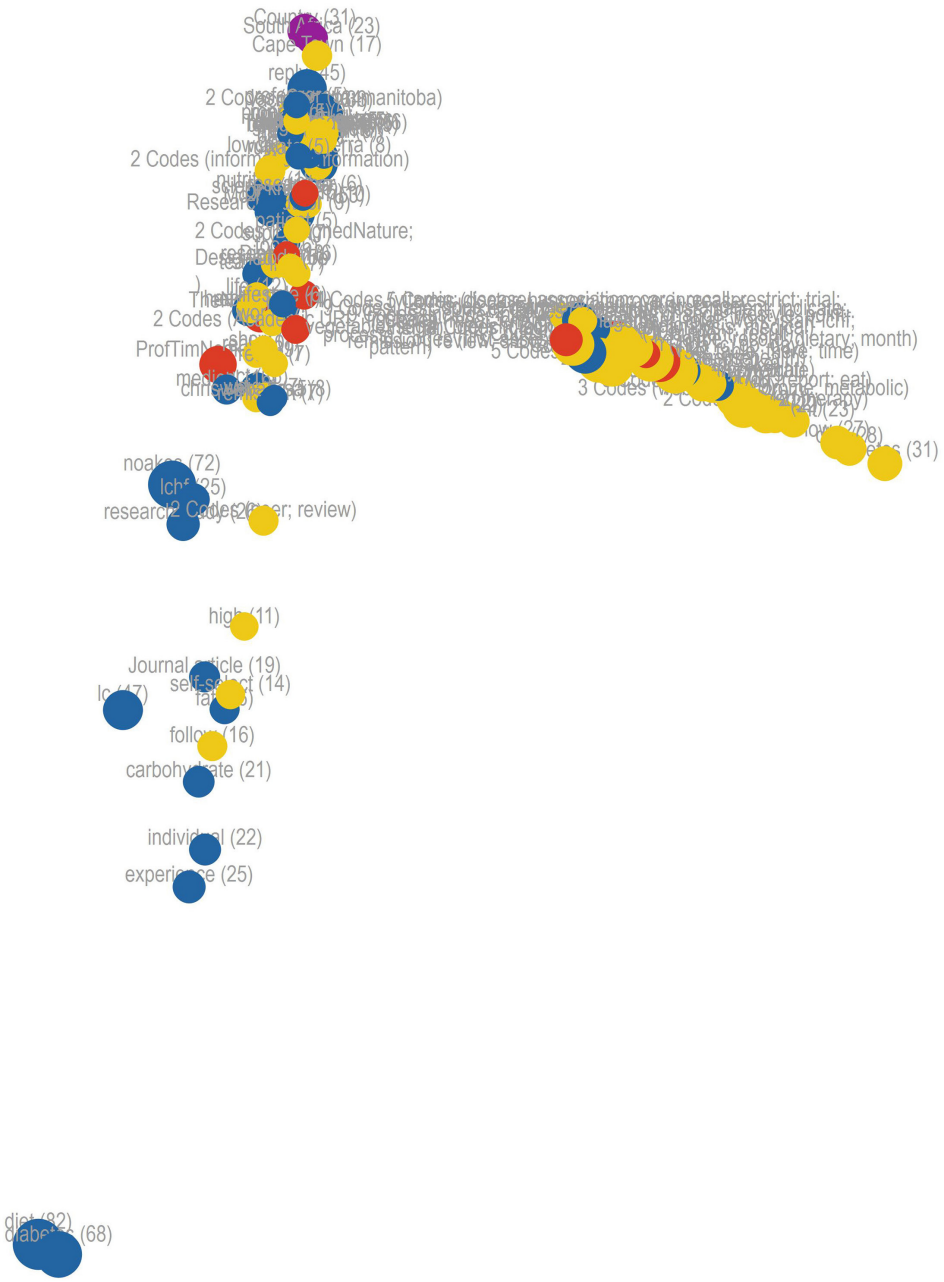
Proximity map for all word frequencies.

A proximity map (Figure 2) shows the most popular words in yellow for Webster et al.'s (2019) article. The most prevalent topics mentioned in tweets are shown in blue, users in red, and their countries in purple. The left-hand side of the proximity map suggests a heavy overlap between Webster et al.'s frequently used terms and the tweet content that users created in sharing and discussing that article. In contrast, the right-hand side features many of the article's terms that were not included in tweets.

### S2 academic links are shared by prominent IR advocates repeatedly

**Figure 3:**
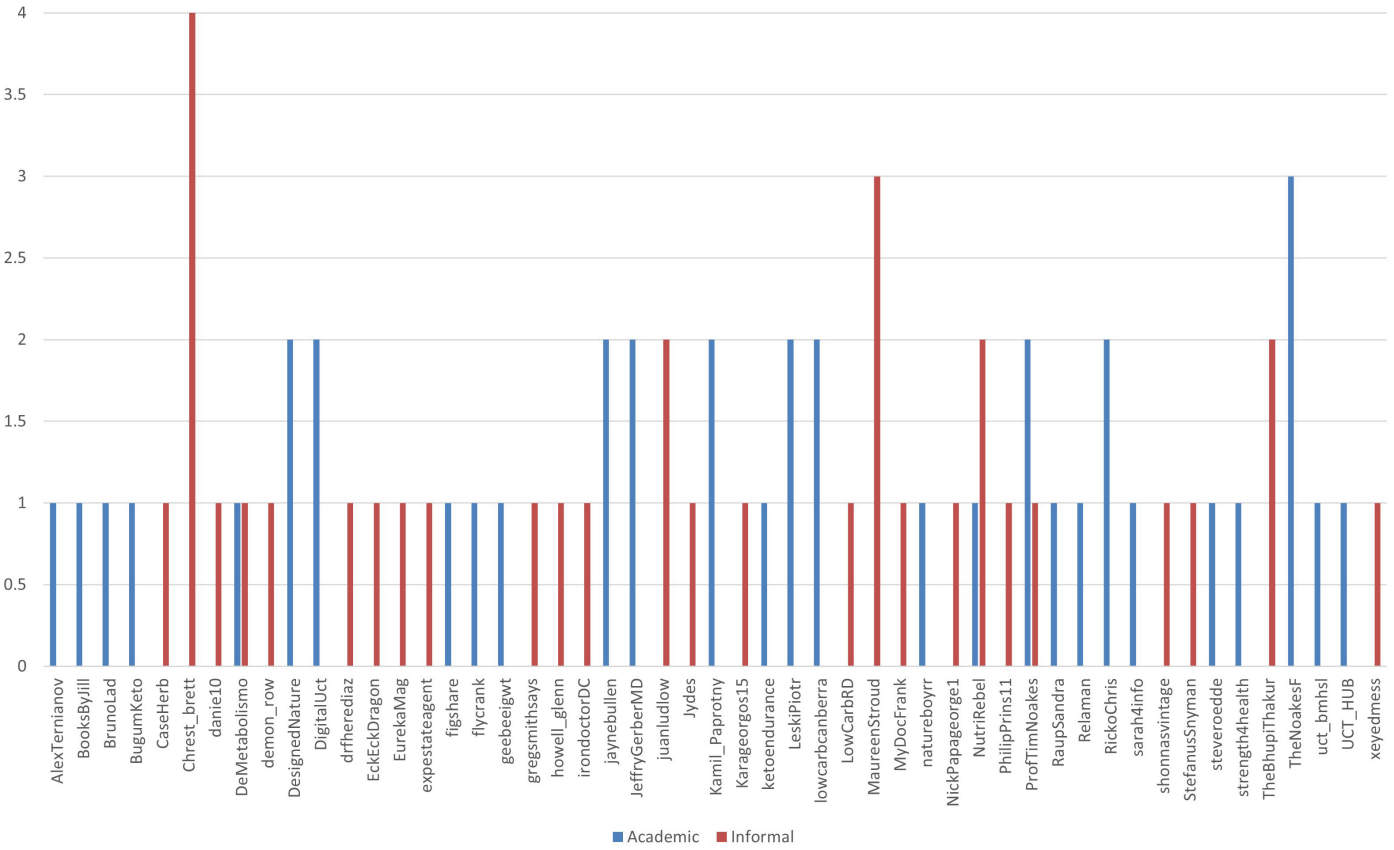
The frequency of contribution by the user is linked to the type of publication's link.

Webster et al. (2019)'s most frequent sharers are shown in Figure 3. (i) Academic links are shown in blue, and (ii) the others in red. Few accounts shared the link more than once or chose to share links from academic publishers and other sources. Amongst the repeat sharers, prominent IR advocacy organizations (such as @LowCarbCanberra) and individuals (like @JeffreyGerberMD) largely posted academic publication links. This reflects the credibility of these sources within the academic use of Twitter for scientific communication.

The inaccurate results for the two outliers are an issue with automated link share reporting. They did not “reshare” the article four and three times, as shown. Instead of representing individual shares, these were erroneously counted multiple times from a seven-tweet thread. An informal link's one-time share became recounted as “seven.”

### S3 most shared by influential Twitter users in England and South Africa

**Figure 4:**
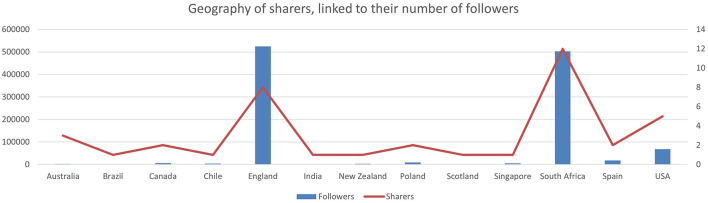
The geography of sharers linked to a tally of their users' followers.

Figure 4 shows that the most influential tweeters and shares of Webster et al. (2019) emanated from England and South Africa. Additionally, users in Australia, Brazil, Chile, Canada, India, New Zealand, Poland, Scotland, Singapore, Spain, and the United States of America shared the article.

### S4 the findings of the multimodal content analysis are corroborated

A relational analysis illustrated the proximity and interconnections between codes.

**Figure 5:**
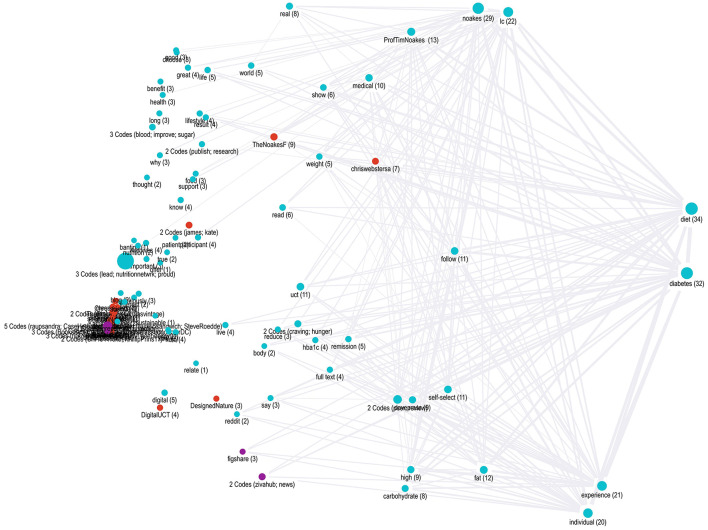
A semantic proximity map shows lines between coded colors.

The analyst produced an intersection, co-occurrence, and proximity map. The proximity maps were the most useful in showing the proximity of codes for tweets across all the spreadsheets. In contrast, the intersection and co-occurrence maps were limited to showing URL-sharing data within individual spreadsheets. Figure 5 shows user profiles in red, tweet content in blue, and locations in purple. A (#) accompanies each legend, denoting its specific code number.

Overall, the semantic proximity map collaborated with the findings of the multimodal content analysis regarding who was involved and what they communicated:

Regarding the content discussed, the content nodes on the right for “diabetes” and “diet” were strongly linked to many others. The terms “weight” and “medical” were tied to the research article's content, as were those that stretch across the bottom, such as “individual,” “self-selected,” and “high fat.” In the map's top left were terms tied to the upsides described in Webster et al.'s (2019) LCHF case studies, such as “improve,” “blood,” and “sugar.”

Figure 5 shows the article's fourth author, Professor Tim Noakes, the most mentioned sharer. His status as an international sport science expert and use of provocative posts linking IR to other scientific controversies help explain his centrality to Twitter engagements. This is similar to how entrepreneurs' provocative tweets on new ventures help drive interaction with their content (Seigner et al., 2023).

### S5 qualitative-led studies are needed to accurately identify sentiment and stance

The statistician initially used Atlas.ti and MAXQDA's automated tools for sentiment analysis. In checking both results, she found many instances where sentiment was incorrectly described as negative where it should have been positive. The following tweet, originally in Portuguese: “*Research by Dr. Tim Noakes achieved total or partial REMISSION (!!) of Type 2 Diabetes in 24 subjects who followed the Ketogenic/Low Carb Diet for 6–35 months, no medication required! Incredible news*” was coded as slightly negative, though the content is positive. This suggested a methodological limitation of quantitative approaches, such as QDAS, in accurately analyzing static Twitter data.

### S6 meta-level critiques of Twitter data sources and results are needed

The SNA uncovered concerns with incorrect automated counts for link shares (in S2) and false results for sentiment analysis (S5). This indicates that microblogging statistics may oversimplify complex categories, leading to inaccurate comparisons. Quantitative simplifications may fail to capture the nuanced complexities of qualitative data. In response, the sixth finding was that a close reading of Twitter data presents a distinct opportunity for meta-critique. Qualitative research can support critiques of microblogging data sources. The results are derived from the automated analyses of data whose categorization is inept.

### S7 identifying issues with QDAS support for static data analysis

While MAXQDA™ works very well with a dictionary for auto-coding, it did not work well with static Twitter data in spreadsheets. Although the dataset was relatively small, the software frequently crashed and operated sluggishly. In contrast, there was no automatic coding of words option in Atlas.ti. It does not support the required dictionary. Consequently, the statistician found that (lack of) QDAS support for static Twitter data analysis could be a concern.

### Development of meta-inferences

The development of meta-inference is the final step in a mixed-methods research project (Schoonenboom, 2022). Following Younas et al. (2023), a seven-step process (i–vii) was followed for developing meta-inferences from findings. The work on the first stages (steps I, II, III, and V) is shown in Table 3 and (step vi) in Table 4. The researchers skipped step iv because comparing the various types of qualitative and quantitative inferences was irrelevant to the main research question's qualitative emphasis.

**Table 3:** Inferences from a semantic network and a multimodal content analysis.

**Step**	**Process**
**I**	**Knowledge-based inferences for the role that qualitative research can play with small data**
1.	Qualitative research can support an understanding of micro-bloggers' identity work.
	(Describe semiotic strategies in the identity work of Twitter profiles.)
2.	Qualitative research supports the understanding of micro-level online practices in research article sharing.
	(Describe a wide variety of Twitter users' multimodal practices in sharing a science article.)
3.	Qualitative research can support a sociological understanding of a communication event's context.
	(Situate micro-level communications within a broader meso- and macro-level context.)
4.	Qualitative research can describe how promoting a science article as state-of-the-art news may relate to broader debates in the field.
	(A cultural sociology of scientific knowledge approach can link scholars' sharing of articles to the debates within their field.)
5.	Qualitative research can code the types of conversations that microbloggers have.
	(Discourse analysis can situate threads as consensual, discursive, or confrontational.)
6.	Qualitative research can define whether science threads are pro-social or anti-social.
	(Cyber harassment and discourse analysis lenses can suit identifying pro-social conversations.)
7.	Qualitative research can uncover insights about the relationships between micro-bloggers.
	(An SNA can explore the links between Twitter sharers of scientific content, such as their countries.)
8.	Qualitative research can uncover relationships and cultural nuances within microblogging networks.
	(The qualitative aspects of an SNA can uncover relational patterns.)
**II**	**Experience-based inferences for qualitative research's potential role**
1.	Health science scholars find value in sharing their latest scientific publications via Twitter.
	(Code C. Twitter user self-presentation and attitude to Cholesterol vs.IR paradigm.)
2.	Although such sharing is a popular practice for self-promotion, little is known regarding the varied practices that scholars and other micro-bloggers use in sharing science articles.
	(Code D. Practices while sharing the article)
3.	Health scholars and allied professionals use popular social media platforms to network for the emergent IR paradigm.
	(Code E. Communication topics in conversation threads)
4.	Advocates for the IR paradigm have pro-social thread discussions with its critics. However, such debate can be controversial
	(Code F. Examples of informal debate or cyber harassment)
**III**	**Data-driven semantic network analysis inferences regarding Twitter users' practices**
S1.	Twitter communications are closely linked to the content of Webster et al. (2019).
S2.	Academic links are shared by prominent IR advocates repeatedly.
S3.	Twitter content on Webster et al. (2019) was most shared by users in England and South Africa.
S4.	The findings of the multimodal content analysis have been corroborated.
S5.	Qualitative-led studies are needed to accurately identify sentiment and stance.
S6.	Meta-level critiques of Twitter data sources and automated results are needed.
S7.	Identify issues with QDAS support for static data analysis
**IV**	**Data-driven multimodal content analysis inferences regarding Twitter users' practices**
M1.	Professionals interested in promoting the IR paradigm largely shared the science article.
M2.	Critics replied to tweets rather than sharing links to the science article.
M3.	Users followed many practices when sharing news about a science article.
M4.	The conversational threads about the article were pro-social, even if antagonistic.
**V**.	**Eliminating speculative inferences**
1.	The findings of the multimodal content analysis have been corroborated (repetition of M1-M4).
2.	Qualitative-led studies are needed to accurately identify sentiment and stance (covered by S6).

**Table 4:** Joint display of inferences and meta-inferences for analyses of a science article's sharing on Twitter.

**Overarching themes**	**SNA results**	**MCA results**	**Meta-inferences**
Twitter users who shared a science article's URL	[1] South African and UK English Twitter users most often shared Webster et al. (2019) articles and other related links.	[2] Webster et al. (2019)'s article and related news were largely shared by IR and LCHF health professionals	IC [1] IR health professionals most often shared Webster et al. (2019). *Explanation*
Twitter users' practices in sharing a science article's URL	[3] The terms that users mentioned in their posts were closely related to Webster et al. (2019)'s article.	[4] Twitter users followed a myriad of link-sharing practices.	IC [2] Tweet content was closely related to the content of Webster et al. (2019). *Confirmation*
News on Webster et al. (2019) article's sharing by critics	[5] No critics shared URLs for Webster et al. (2019).	[6] Critics replied to tweets rather than sharing links to this science article.	IC [3] While critics commented on this article, they did not promote news. *Explanation*
Twitter discussion of Webster et al. (2019)	[7] Twitter users from many countries shared articles linked to Webster et al. (2019).	[8] No posts featured anti-social behaviors. Many threads featured pro-social communication.	IC [4] The science article was shared in many territories and stimulated pro-social conversations. *Explanation*
Concerns with results from Twitter data	[9] Meta-level critiques of Twitter data sources and results are needed.	[10] Twitter data was benchmarked against Altmetrics, and missing data was added.	IC [5] It is challenging to do an accurate Twitter sentiment or analysis of “healthy conversations” *Contradiction*
Concerns with the support of qualitative tools for data analysis with static Twitter data	[11] Atlas.ti could not support the type of SNA that the statistician wanted to do. Analysis via MAXQDA with a small spreadsheet proved difficult.	[12] Multimodal content analysis requires that results in static spreadsheets be checked in Twitter- certain modes (such as imagery, emojis, and videos) are lost in spreadsheet translation.	IC [6] Some types of qualitative analysis with static Twitter data may not be well-supported by QDAS tools *Contradiction*

[#], claim; IC [#], integrated claim.

In the initial stages of this article's fieldwork, the study adopted a multimodal content analysis strategy, followed by a semantic network one. The claims from these mono-methods are presented in Table 3's rows (iii) and (iv). The analysts then elaborated on the meta-inferences from the small data project step-by-step before eliminating the repetitive speculative inferences of row (v). Subsequently, a meta-inference confirmation determines the alignment between different sets of data in a research study to establish if they confirm or potentially expand each other.

Table 4 shows the points of explanation, confirmation, and contradiction that were established by comparing the analysts. The meta-inferences are listed in [brackets]. These were derived from the comparison and largely explained or confirmed the mono-methods' findings.

Two important contradictions emerged in the research process itself. First, there was mis-categorization by quantitative tools in their automated sentiment analysis and inaccurate article share tallying from threads. The researchers flagged that such inaccurate results can be spotted through meta-level critiques. This flags the need for an accurate description of categories based on rigorous qualitative research for apt categorization around meaning(s). Second, while QDAS tools are marketed as making Twitter analysis efficient, the statistician faced challenges using small static spreadsheets or being unable to auto-code depending on the QDAS she worked with.

#### Data availability

The raw data supporting the conclusions of this article will be made available by the authors without undue reservation.

#### Ethics statement

##### Human subject research

The studies involving humans were approved by the Cape Peninsula University Faculty of Health and Wellness Sciences Research Ethics Committee. The studies were conducted in accordance with the local legislation and institutional requirements. Written informed consent for participation was not required from the participants or the participants' legal guardians/next of kin in accordance with the national legislation and institutional requirements.

##### Human images

Written informed consent was not obtained from the individual(s) to publish any potentially identifiable images or data in this article because this information is in the public domain and is not controversial. Sharing pseudonymized tweets in an article does not place their authors in a negative light, so it holds no risk to authors' reputations.

## Ethics

Our research follows the “Ethical decision-making and Internet research (version 2.0) recommendations of the American Association of Internet Researchers” (AAoIR) Ethics Working Committee (Markham et al., 2012) and AAoIR's initial guidance (Ess and Jones, 2004). The ethics committee at the Cape Peninsula University of Technology's (CPUT) Faculty of Health and Wellness Sciences reviewed and approved our project's process and this output (2024URI_NEG_002). Our project complies with Twitter's criteria for data use.

Micro-bloggers are unlikely to expect a review of their tweets for research purposes. Most users expect to give their consent before their tweets are researched (Fiesler and Proferes, 2018). This concern must be weighed against the considerable effort required to provide full anonymity. The authors decided against full anonymization since it would reduce the richness of this study's illustrative examples. At the same time, while there appear to be no reputational risks in sharing tweeters' examples, this study does not show who authored particular tweets.

## Discussion

The findings from each mono-method and their combined meta-inferences suggest the potential contributions that qualitative research can make to studies that focus on small data from microblogging communications.

Qualitative research methods can uncover meanings from inside a communication phenomenon. These methods support explorations of who chooses to tweet scientific content, a detailed description of their practices, and how these may link back to individuals' identity work. A novel finding spotlighted the wide variety of users' sharing practices. Almost 20 different types were uncovered.

Qualitative research can provide a rich contextual framing for how micro-practices relate to important social dynamics. A sociological framing situates Webster et al.'s (2019) publication as a communication event within a long-running debate in the health sciences. Since their article motivated the emergent IR paradigm, its sharing presented opportunities for health professionals to do supportive identity work. As the LCHF interventions they prescribe draw on the IR paradigm, sharing its positive research developments would assist their professional credibility while growing their visibility to sympathetic networks and potential customers. In contrast, the article's critics did not promote its links, choosing to engage in pro-social threads with IR/LCHF proponents.

A qualitative process supported the identification of a wide variety of pro-social practices amongst Twitter sharers by Webster et al.'s (2019) study. This contrasts with quantitative scholarship reporting on the negative practices in Twitter shares for popular dentistry articles. Monomania underpinned their high tweet counts, with most tweeting being mechanical, seemingly devoid of human thought (Robinson-Garcia et al., 2017). Many duplicate tweets originated from centralized management accounts or bots. Few tweets represented genuine engagement with the shared articles, and very few were part of conversations. The contrast with the pro-social Webster et al. (2019) suggest the need to contextualize micro-level communications within higher-level social strata, such as the Global Health Science field and its key debates.

Qualitative research focusing on an in-depth exploration of small data can contribute to the research process by supporting meta-level critiques of missing data, (mis-) categorizations, and flawed automated (and manual) results.

## Conclusion

This study contributes a rationale for the contribution of qualitative methods in researching with Twitter and other microblogging data. The study compared two qualitatively led analyses regarding the unusual topic of tweets that shared links to a science article in support of the emergent IR paradigm. A multimodal content analysis supported the rich-and-thick description of users' Twitter practices, showing that they have a much greater variety than previously described in the SciComm literature. This analysis revealed that many of these practices were related to users' professional contexts and digital identity work. Many health professionals' promotion of LCHF lifestyles seemed favored by the article's findings on IR. A semantic network analysis confirmed that communications related to the article, with proponents and critics having very different responses. While prominent proponents shared links repeatedly, critics never did, choosing to reply. The SNA flagged issues with the automated identification of sentiment, suggesting that qualitative research can contribute to accurately categorizing communicators' stances. Meta-inferences from both analyses suggested the important role of qualitative methods for supporting critiques of Twitter data, automated QDAS functions, and verifying the definition of categories that underpin automated results.

## Strengths and weaknesses

This research exhibits the eight “big tent” strengths expected for high-quality qualitative research (Tracy, 2019): It makes a (1) timely and interesting *contribution* to a neglected research subject by proposing a rationale for qualitative approaches to microblogging research with small data. The two research lenses of multimodal content and SNA were (2) *rigorously* applied; the meta-inference comparison supports the credibility of the research findings through coherence and expansion. Simultaneously, an in-depth review of the data on which the analysis was based helped ensure that key practices by micro-bloggers did not disappear due to Twitter data export and QDAS import cleaning practices. A conventional approach might only focus on English tweets. This would miss the valuable role of science promoters making translations for their non-English-speaking followers.

Furthermore, adding memos for translated tweets proved useful for incorporating a wider range of contributors. A similar issue emerged with sentiment analysis, where the researcher's knowledge of the text's content differed from MAXQDA's automated findings. Given that the positive, neutral, and negative results seemed inaccurate, they were excluded.

This study's manuscript evolved over a 4-year period. The authors are (3) *sincere* in sharing the challenges they experienced during the project. They are transparent in the phases and steps taken to prepare their analyses. The (4) *credibility* of the research is supported by both thick descriptions and the crystallization resulting from meta-inference development. An element of multivocality drew on researchers' perspectives with differing professional backgrounds: a statistician, a media studies pragmatist, an information technology qualitative analyst, and a transdisciplinary research expert. These researchers differed in their opinions on the value of academic Twitter but agreed that this study's findings would (5) *resonate* with other scholars. This study's findings are transferable to microblogging scholars who could benefit from learning about the roles of qualitative research in analyzing small data. This manuscript's organization and the aesthetic merit in its well-designed figures and tables should support resonance with readers. While the results of the Twitter analysis are not generalizable, a similar methodology could yield valuable insights into the sharing of other popular articles.

The study's topic has methodological (6) *significance* in assisting its readers in understanding how to analyze a science article's shares on a microblogging platform. The study has conceptual significance in building theory by presenting a fresh rationale. Suggestions for future research in the next section may contribute to heuristic significance by encouraging scholars to address the gaps it spotlights. As described under (7) *Ethics*, this project was covered by an institutional review. This study is (8) *meaningfully coherent* in following a clear line of inquiry. Its research question links the literature reviewed to the article's foci, methods, and findings.

Regarding weaknesses, the research team could only focus on learnings produced from two qualitatively led methods. Many other feasible approaches to researching small data, such as critical discourse analysis or grounded theory, could be explored.

## Future research

There are many opportunities to build on this study's contributions. Researchers could explore and refine the role of qualitative methods in analyzing microblogging communications for science articles. Qualitative research that adds data outside small data microblogging extracts could strengthen the study's rationale.

While this study focused on a relatively small communication event, many examples of meaningful exchanges materialized, indicating the importance of studying events within long-running scientific debates. This resonates with Díaz-Faes et al.'s (2019) suggestion for the scholarship that explores Twitter shares by specific scientific communities. By focusing on tweet shares, our research contributes to the great imbalance in Twitter's use as a social media platform, which is over-researched (Tufekci, 2014). To combat this, scholars can explore science sharing via microblogging platforms outside the Anglosphere. Non-platform-specific creative situational approaches are needed since these provide a much-needed understanding of wider platform dynamics (Özkula et al., 2022).

## Data Availability

The raw data supporting the conclusions of this article will be made available by the authors, without undue reservation.

## Footnotes

^1^Twitter was purchased by Elon Musk in October 2022. As part of his plans for expanding this digital platform to an “everything app,” Twitter was rebranded to “X” in late July 2023. Our research article refers to Twitter throughout to reflect that we report on “tweets” between late 2019 and the end of March 2021 from the “old” platform. Technically, these data extracts were not from an “X platform.” Referring to “Twitter” enables the authors to accurately speak to pre-August 2023 “Twitter research” in science communication. Attempting to term such scholarship as “X research” may well confuse readers by potentially conflating contemporary research into the X platform with scholarship done on Twitter that stretches back until 2007.
